# Supplementary material for: Regeneration in the absence of canonical neoblasts in an early branching flatworm
Source: Nat Commun. 2025 Jan 31;16:1232. doi: 10.1038/s41467-024-54716-x (PMC11785736; doi:10.1038/s41467-024-54716-x)
Supplement: Supplementary file 1 — Supplementary Information [file 41467_2024_54716_MOESM1_ESM.pdf]

Regeneration in the absence of canonical neoblasts in an early branching flatworm

Ludwik Gąsiorowski[1], Chew Chai[2], Andrei Rozanski[1], Gargi Purandare[1], Fruzsina Ficze[1], Athanasia Mizi[3], Bo Wang[2], Jochen C. Rink[1, *]

[1] Department of Tissue Dynamics and Regeneration, Max Planck Institute for Multidisciplinary Sciences, Göttingen, Germany
[2] Department of Bioengineering, Stanford University, Stanford, USA
[3] Institute of Pathology, University Medical Centre Göttingen, Göttingen, Germany
[*] Corresponding author: jochen.rink@mpinat.mpg.de

Supplementary Figures 1 – 6

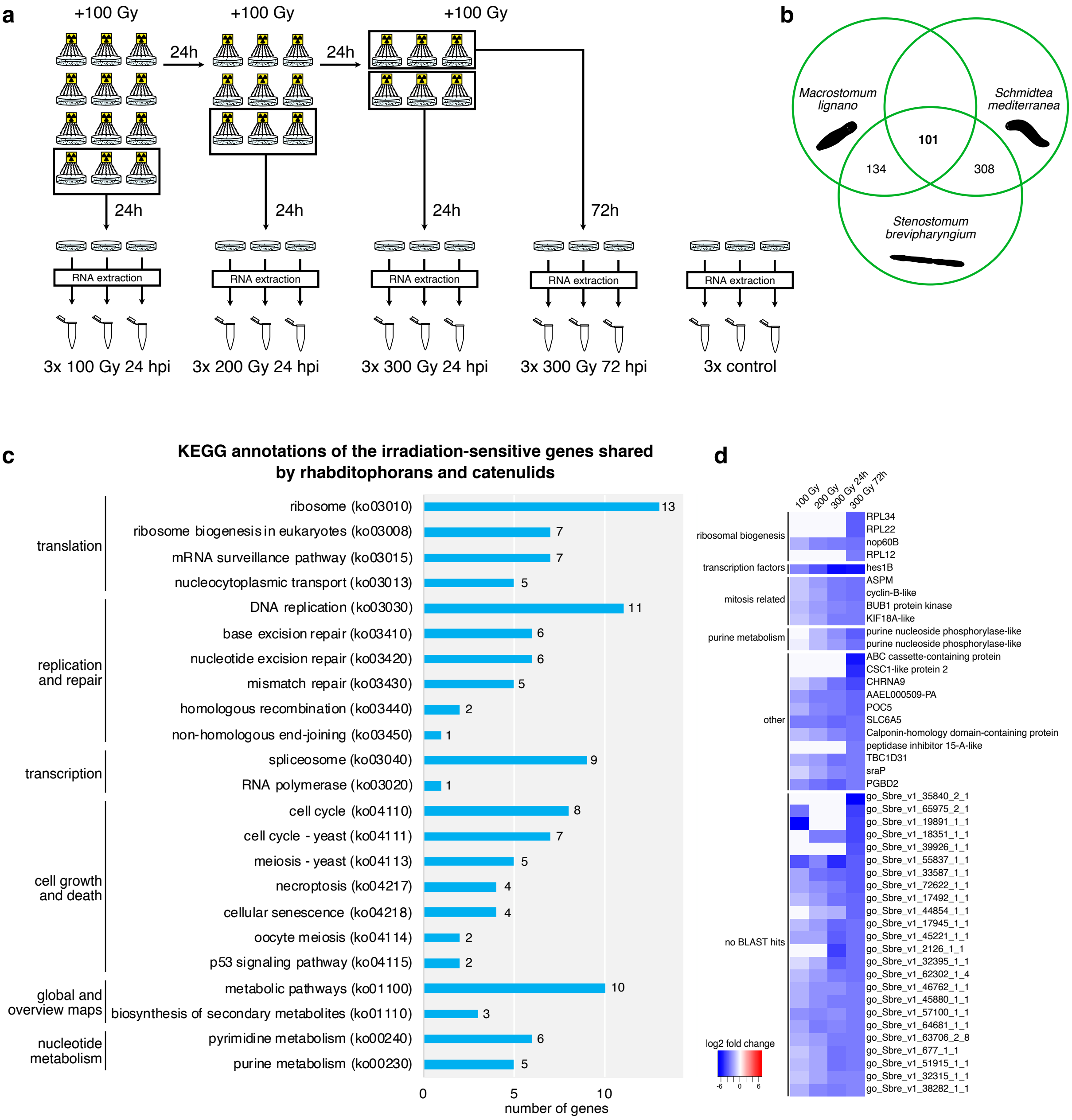

Supplementary Fig. 1. Transcriptomics of the irradiated worms allow detailed studies of the irradiation-sensitive genes in *Stenostomum brevipharyngium*. a, experimental design of the bulk RNA sequencing of irradiated worms. **b**, number of the irradiation-sensitive orthologs shared between *S. brevipharyngium*, and two rhabditophorans – *Macrostomum lignano* (Macrostomida) and *Schmidtea mediterranea* (Tricladida). **c**, KEGG annotations of the 101 irradiation-sensitive genes shared between all three species. **d**, a heatmap of the most down-regulated genes in lethally irradiated *S. brevipharyngium* (>10-fold change at the 300 Gy 72 hpi), gray boxes indicate insignificant changes (adjusted p-value<0.05, two-sided Wald test adjusted with the procedure of Benjamini and Hochberg). To the right, the BLAST annotations are provided for each gene (when possible), while the left column contains the putative cellular function of the genes.

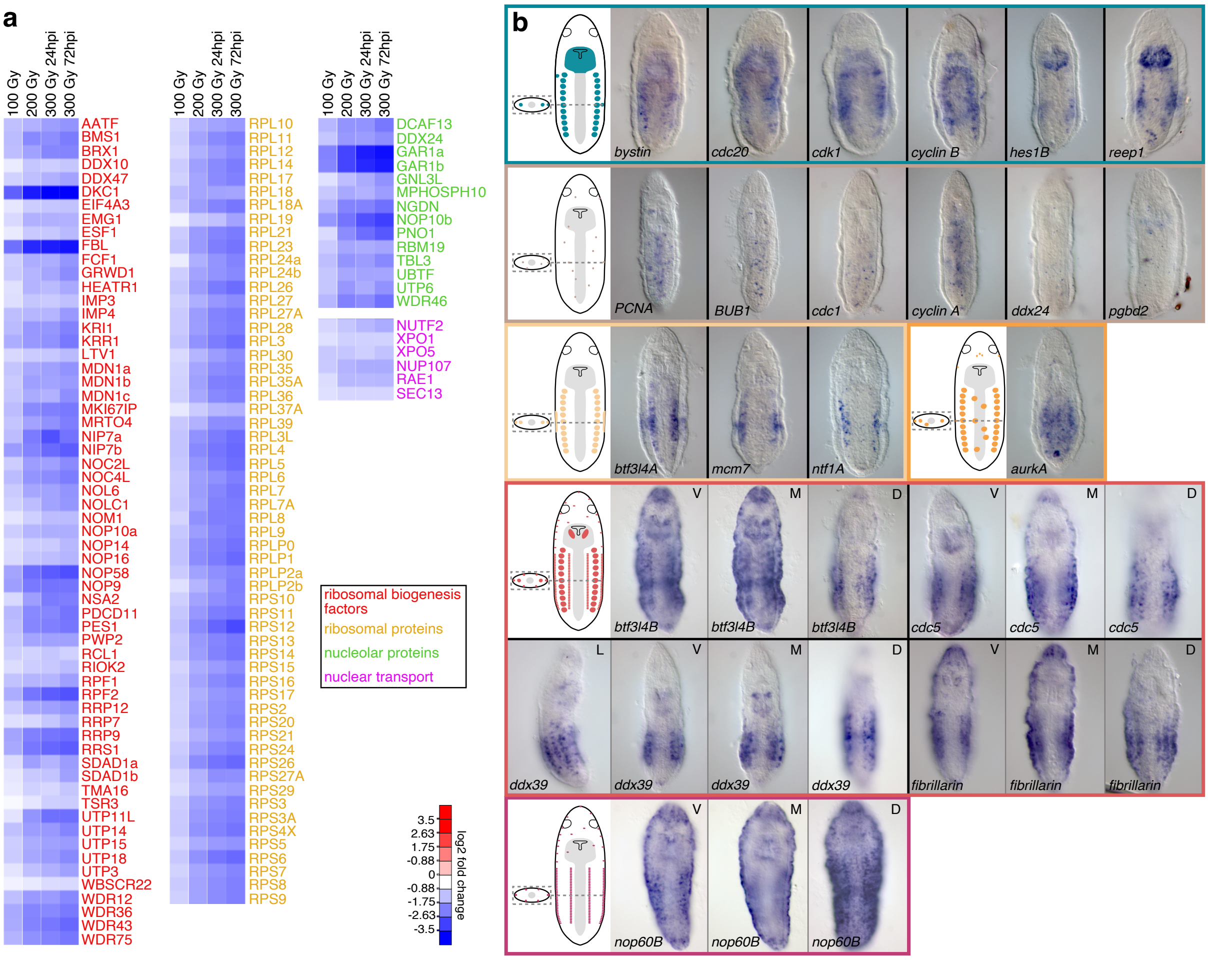

Supplementary Fig. 2. Analysis of the irradiation-sensitive genes in *Stenostomum brevipharyngium*. **a**, a heatmap showing systematic downregulation of the genes involved in ribosomal biogenesis. **b**, expression patterns of the selected irradiation-sensitive genes, the genes are grouped by the common expression patterns, indicated on the cartoons. L, lateral view; V, ventral surface; M, midsection; D, dorsal surface.

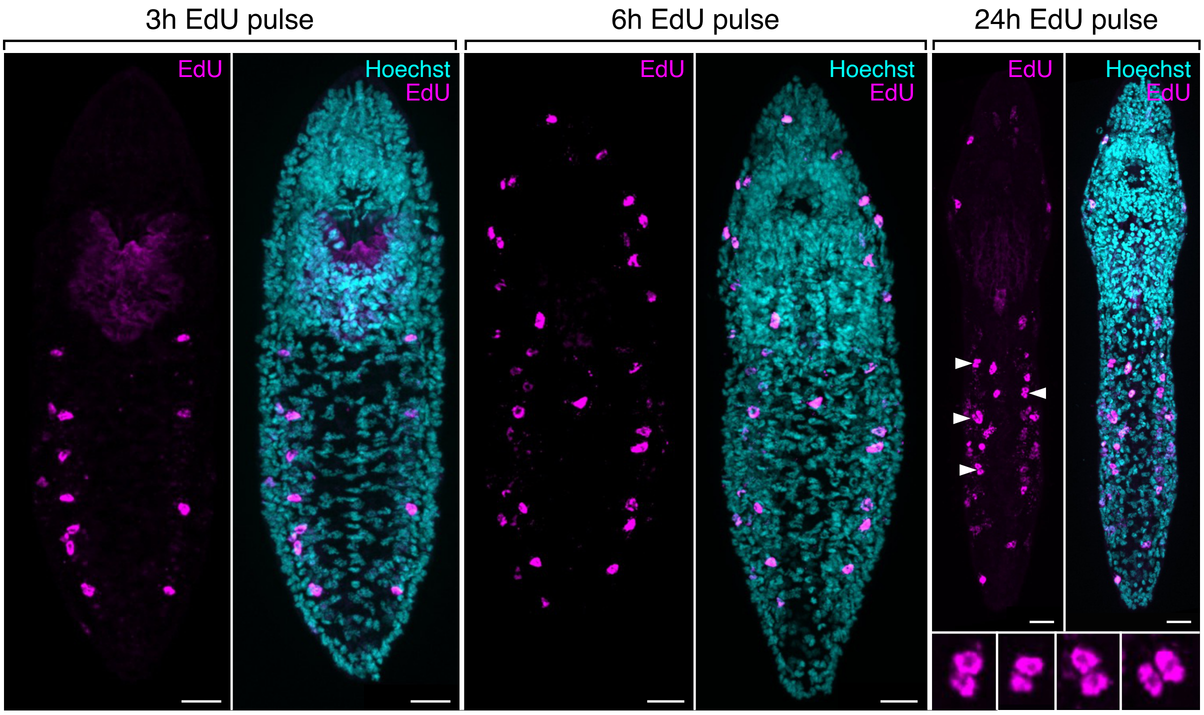

Supplementary Fig. 3. EdU incorporation into mitotically active cells in S-phase. Scale bars represent 10 µm. Arrowheads indicate EdU^{+} cell doublets (enlarged on insets).

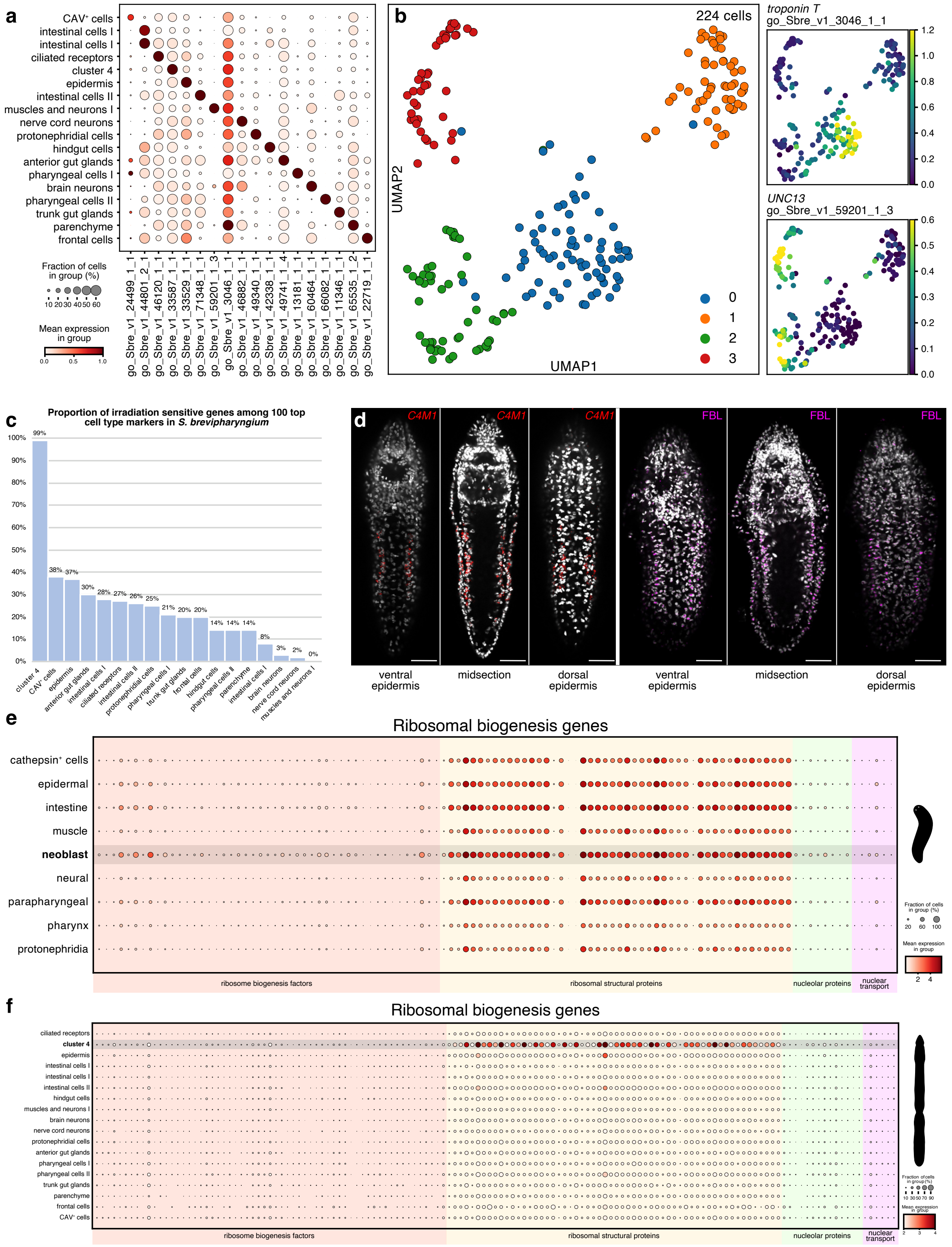

Supplementary Fig. 4. Details of the single-cell transcriptomic analysis. a, dotplot showing the specificity of molecular markers used to detect retrieved cell types. **b**, sub-clustering of cluster 7 into troponin-positive muscular cluster (subcluster 0) and UNC-13-positive neural cluster (subclusters 2 and 3). **c**, Irradiation sensitivity of particular cell type clusters. **d**, expression of the *cluster 4 marker 1* (red) in deep stem cells along the gut, and longitudinal bands of cells in ventral, lateral, and dorsal epidermis follows staining with antibody against nucleolar marker fibrillarin (magenta), which is present in the irradiation-sensitive cells. **e–f**, dotplots showing cell type-specific expression of the genes involved in ribosomal biogenesis in *Schmidtea mediterranea* (**e**) and *S. brevipharyngium* (**f**).

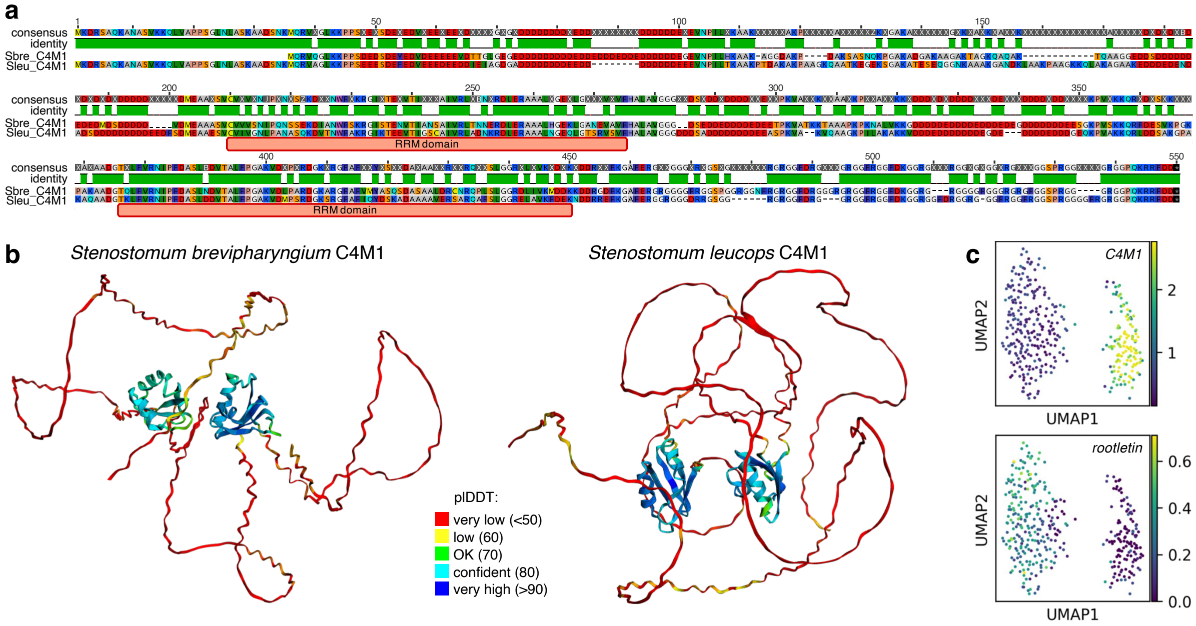

Supplementary Fig. 5. C4M1 as a marker for *Stenostomum* stem cells. a, Conservation of the protein encoded by *C4M1* between *S. brevipharyngium* and *Stenostomum leucops*. In both species, the protein sequence contains long stretches of negatively charged amino acids (red residues in the alignment) and two RNA recognition motif domains (RRM). **b**, Alpha-fold predictions of the structure of C4M1 proteins from *S. brevipharyngium* and *S. lecuops*; note the poor prediction quality over most of the sequence (red/orange color shades), but high confidence prediction of both RRM domains in both species (blue/green color shades). **c**, subclustering of cluster 4 reveals the presence of two cell populations within the cluster – one showing elevated expression levels of *C4M1* and the other positive for the epidermal marker *rootletin*.

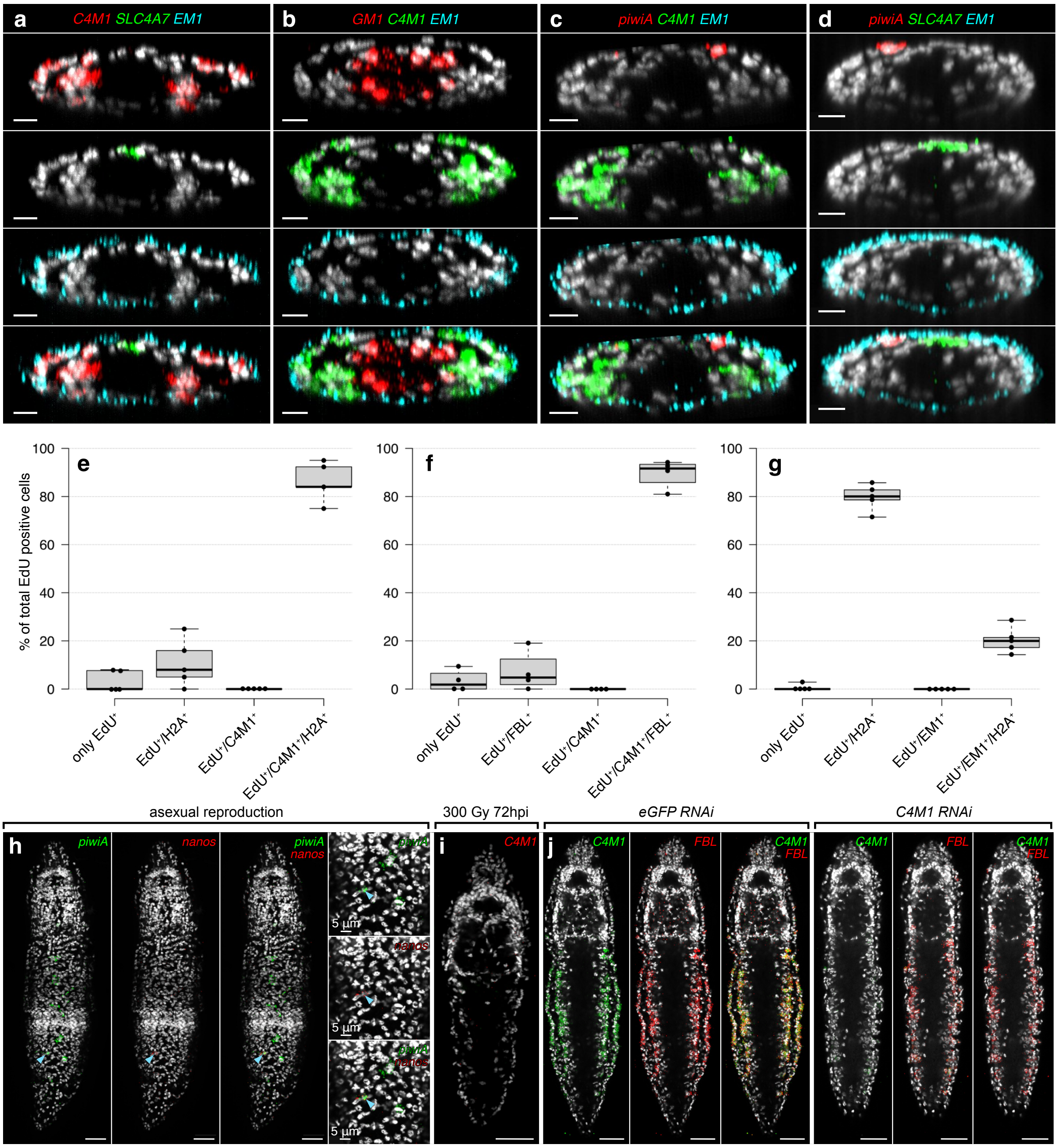

Supplementary Fig. 6. Anatomical distribution of the stem and germline cells of *Stenostomum brevipharyngium*. a–d, Confocal Z-sections at the mid-trunk level, labelled with the stem cell marker (*C4M1*; a,b) or the germ-line marker (*piwiA*; c,d) in combination with the indicated tissue markers (*SLC4A7*: protonephridium; *EM1*: epidermis; *GM1*: intestine). Scale bars represent 5 µm. **e–g Colocalization between** a 2h EdU pulse and the indicated stem cell and epidermal markers. The frequencies of the indicated marker combinations are plotted as a percentage of all EdU$^+$ cells, center lines show the medians, box limits indicate the 25th and 75th percentiles, and whiskers extend to minimum and maximum values. **h**, *nanos* expression in a single *piwiA*$^+$ cell cluster (cyan arrowhead) of a specimen in the process of asexual reproduction. Scale bars represent 20 µm, if not indicated otherwise. **i**, absence of *C4M1*$^+$ cells in the midsection of a lethally irradiated specimen, scale bar represents 20 µm. **j**, efficiency and specificity of *C4M1 RNAi*, validated by HCR in situ hybridization, scale bar represents 20 µm. Source data for panels **e–g** are provided as a Source Data file.
