# Supplementary material for: Regeneration in the absence of canonical neoblasts in an early branching flatworm
Source: Nat Commun. 2025 Jan 31;16:1232. doi: 10.1038/s41467-024-54716-x (PMC11785736; doi:10.1038/s41467-024-54716-x)
Supplement: Supplementary file 3 — Description of Additional Supplementary Files [file 41467_2024_54716_MOESM3_ESM.pdf]

Description of Additional Supplementary Files

File Name: Supplementary Data 1
Description: Differen@ally expressed genes in irradiated *Stenostomum brevipharyngium*

File Name: Supplementary Data 2
Description: Comparison of irradia@on-sensi@ve genes in *Stenostomum*

File Name: Supplementary Data 3
Description: IDs of genes used for RNA in situ hybridization

File Name: Supplementary Data 4
Description: Manually selected markers for single nuclei clusters

File Name: Supplementary Data 5
Description: Manually selected markers for subclusters of cluster 7 (muscles and neurons I)

File Name: Supplementary Data 6
Description: List of *Stenostomum* genes supporting grouping of cluster 4 and planarian neoblast in SAMap analysis

File Name: Supplementary Data 7
Description: Changes in the number of individuals and statistical analysis of the irradiation survival curves

File Name: Supplementary Data 8
Description: IDs of genes involved in cell division, ribosomal biogenesis, and germline multipotency program used in this study

File Name: Supplementary Data 9
Description: Buffers used for nuclear extraction

File Name: Supplementary Data 10
Description: Changes in the number of individuals and statistical analysis of the RNAi survival curves

File Name: Supplementary Data 11
Description: Statistical analysis of the regeneration success rate in RNAi experiments
