# Supplementary material for: Regeneration in the absence of canonical neoblasts in an early branching flatworm
Source: Nat Commun. 2025 Jan 31;16:1232. doi: 10.1038/s41467-024-54716-x (PMC11785736; doi:10.1038/s41467-024-54716-x)
Supplement: Supplementary file 5 — Reporting Summary [file 41467_2024_54716_MOESM5_ESM.pdf]

nature portfolio

Corresponding author(s): Jochen Rink

Last updated by author(s): Oct 24, 2024

Reporting Summary

Nature Portfolio wishes to improve the reproducibility of the work that we publish. This form provides structure for consistency and transparency in reporting. For further information on Nature Portfolio policies, see our Editorial Policies and the Editorial Policy Checklist.

Statistics

For all statistical analyses, confirm that the following items are present in the figure legend, table legend, main text, or Methods section.

n/a	Confirmed	
☐	☒	The exact sample size (*n*) for each experimental group/condition, given as a discrete number and unit of measurement
☐	☒	A statement on whether measurements were taken from distinct samples or whether the same sample was measured repeatedly
☐	☒	The statistical test(s) used AND whether they are one- or two-sided<br>*Only common tests should be described solely by name; describe more complex techniques in the Methods section.*
☒	☐	A description of all covariates tested
☒	☐	A description of any assumptions or corrections, such as tests of normality and adjustment for multiple comparisons
☒	☐	A full description of the statistical parameters including central tendency (e.g. means) or other basic estimates (e.g. regression coefficient) AND variation (e.g. standard deviation) or associated estimates of uncertainty (e.g. confidence intervals)
☒	☐	For null hypothesis testing, the test statistic (e.g. *F*, *t*, *r*) with confidence intervals, effect sizes, degrees of freedom and *P* value noted<br>*Give P values as exact values whenever suitable.*
☒	☐	For Bayesian analysis, information on the choice of priors and Markov chain Monte Carlo settings
☒	☐	For hierarchical and complex designs, identification of the appropriate level for tests and full reporting of outcomes
☒	☐	Estimates of effect sizes (e.g. Cohen's *d*, Pearson's *r*), indicating how they were calculated

Our web collection on statistics for biologists contains articles on many of the points above.

Software and code

Policy information about availability of computer code

Data collection	no software was used to collect the data
Data analysis	FastQC (0.11.9)<br>trimgalore (0.6.6)<br>Trinity suite (v 2.14.0)<br>DESeq2 (v Bioconductor 3.15)<br>Transdecoder (v 5.5.0)<br>orthofinder (v 2.5.4)<br>UMI-tools (1.1.2)<br>cuadapt (4.3)<br>bowtie2 (v2.5.1)<br>SoupX (1.6.2)<br>SAM version 1.0.1<br>Fiji (2.3.0)<br>BoxPlotR<br>SAMap version 1.0.15

For manuscripts utilizing custom algorithms or software that are central to the research but not yet described in published literature, software must be made available to editors and reviewers. We strongly encourage code deposition in a community repository (e.g. GitHub). See the Nature Portfolio guidelines for submitting code & software for further information.

Data

Policy information about availability of data

All manuscripts must include a data availability statement. This statement should provide the following information, where applicable:

- Accession codes, unique identifiers, or web links for publicly available datasets
- A description of any restrictions on data availability
- For clinical datasets or third party data, please ensure that the statement adheres to our policy

Data availability
The raw RNAseq reads on which the S. brevipharyngium reference transcriptome is based have been deposited at the NCBI Sequence Reads Archive (BioProject ID PRJNA1004231). The assembled reference transcriptome is available at the Zenodo.org data repository (doi: 10.5281/zenodo.8239273). The raw RNAseq reads of irradiated worms have been deposited at the NCBI Sequence Reads Archive as BioProject PRJNA1149834. The single-cell transcriptomic data has been deposited iat the NCBI under accession number PRJNA1156255. All microscopy data included with this manuscript are available upon request from the corresponding author.
Source Data are provided with this paper.

Code availability
There is no custom code or mathematical algorithm developed for this study.

Research involving human participants, their data, or biological material

Policy information about studies with human participants or human data. See also policy information about sex, gender (identity/presentation), and sexual orientation and race, ethnicity and racism.

Reporting on sex and gender	n/a
Reporting on race, ethnicity, or other socially relevant groupings	n/a
Population characteristics	n/a
Recruitment	n/a
Ethics oversight	n/a

Note that full information on the approval of the study protocol must also be provided in the manuscript.

Field-specific reporting

Please select the one below that is the best fit for your research. If you are not sure, read the appropriate sections before making your selection.

☒ Life sciences ☐ Behavioural & social sciences ☐ Ecological, evolutionary & environmental sciences

For a reference copy of the document with all sections, see nature.com/documents/nr-reporting-summary-flat.pdf

Life sciences study design

All studies must disclose on these points even when the disclosure is negative.

Sample size	For survival curves 8-24 individuals were included for each replicate within experiment. For cell counting 4-16 individuals were used (depending on the number of available material).
Data exclusions	No data was excluded from the analyses
Replication	Each experiment was performed in at least 2 technical replicates
Randomization	The animals were chosen at random from laboratory cultures
Blinding	Blinding was not possible as experiments were performed solely by one investigator. However, tere was no need for blinding as obtained data are numerical and can be retrieved from the microcopic data available upon request from corresponding author.

Reporting for specific materials, systems and methods

We require information from authors about some types of materials, experimental systems and methods used in many studies. Here, indicate whether each material, system or method listed is relevant to your study. If you are not sure if a list item applies to your research, read the appropriate section before selecting a response.

Materials & experimental systems

n/a	Involved in the study
☐	☒ Antibodies
☒	☐ Eukaryotic cell lines
☒	☐ Palaeontology and archaeology
☐	☒ Animals and other organisms
☒	☐ Clinical data
☒	☐ Dual use research of concern
☒	☐ Plants

Methods

n/a	Involved in the study
☒	☐ ChIP-seq
☒	☐ Flow cytometry
☒	☐ MRI-based neuroimaging

Antibodies

Antibodies used	mouse anti-tyrosinated tubulin primary antibodies, Sigma T9028<br>goat anti-mouse secondary antibodies conjugated with Alexafluor488, Thermo Fisher A-11001<br>mouse anti-Fibrillarin primary antibodies, Thermo Fisher MA316771<br>rabbit anti-Histone H3 phospho S10+T11, abcam ab32107<br>goat anti-rabbit secondary antibodies, conjugated with Alexafluor647, Thermo Fisher A-21244
Validation	Specificity of primary antibodies was not explicitely tested, however, those antibodies has been demonstrated by the producer to show specificity in variety of species from yeast to mouse. Based on the fact that the the epitopes for those antibodies are highly conserved among animals we can assume its specificity in tested flatworm species.<br><br>Specificity of secondary antibodies was tested by inclusion of negative samples in which primary antibodies were omitted.

Animals and other research organisms

Policy information about studies involving animals; ARRIVE guidelines recommended for reporting animal research, and Sex and Gender in Research

Laboratory animals	We used laboratory cultures of Stenostomum brevipharyngium. The cultures were ordered on-line in 2010 from Connecticut Valley Biological Supply as Stenostomum sp. and since then maintained in the laboratory conditions following providers instructions
Wild animals	no wild animals were used in the research
Reporting on sex	researched organisms are asexual
Field-collected samples	no samples were collected in the field
Ethics oversight	The research on microscopic flatworms is not regulated and therefore no ethic oversight is necessary

Note that full information on the approval of the study protocol must also be provided in the manuscript.

Plants

Seed stocks	N/A
Novel plant genotypes	N/A
Authentication	N/A
